# DNA Polymerases ImuC and DinB Are Involved in DNA Alkylation Damage Tolerance in *Pseudomonas aeruginosa* and *Pseudomonas putida*

**DOI:** 10.1371/journal.pone.0170719

**Published:** 2017-01-24

**Authors:** Tatjana Jatsenko, Julia Sidorenko, Signe Saumaa, Maia Kivisaar

**Affiliations:** Department of Genetics, Institute of Molecular and Cell Biology, University of Tartu, Tartu, Estonia; University of Miami School of Medicine, UNITED STATES

## Abstract

Translesion DNA synthesis (TLS), facilitated by low-fidelity polymerases, is an important DNA damage tolerance mechanism. Here, we investigated the role and biological function of TLS polymerase ImuC (former DnaE2), generally present in bacteria lacking DNA polymerase V, and TLS polymerase DinB in response to DNA alkylation damage in *Pseudomonas aeruginosa* and *P*. *putida*. We found that TLS DNA polymerases ImuC and DinB ensured a protective role against *N*- and *O*-methylation induced by N-methyl-N'-nitro-N-nitrosoguanidine (MNNG) in both *P*. *aeruginosa* and *P*. *putida*. DinB also appeared to be important for the survival of *P*. *aeruginosa* and rapidly growing *P*. *putida* cells in the presence of methyl methanesulfonate (MMS). The role of ImuC in protection against MMS-induced damage was uncovered under DinB-deficient conditions. Apart from this, both ImuC and DinB were critical for the survival of bacteria with impaired base excision repair (BER) functions upon alkylation damage, lacking DNA glycosylases AlkA and/or Tag. Here, the increased sensitivity of *imuCdinB* double deficient strains in comparison to single mutants suggested that the specificity of alkylated DNA lesion bypass of DinB and ImuC might also be different. Moreover, our results demonstrated that mutagenesis induced by MMS in pseudomonads was largely ImuC-dependent. Unexpectedly, we discovered that the growth temperature of bacteria affected the efficiency of DinB and ImuC in ensuring cell survival upon alkylation damage. Taken together, the results of our study disclosed the involvement of ImuC in DNA alkylation damage tolerance, especially at low temperatures, and its possible contribution to the adaptation of pseudomonads upon DNA alkylation damage via increased mutagenesis.

## Introduction

Alkylation DNA damage is ubiquitous and can originate both from normal cellular metabolism and from the exposure to environmental pollutants and other methylating agents. Cellular exposure to simple methylating agents, such as methyl methanesulfonate (MMS) and N-methyl-N'-nitro-N-nitrosoguanidine (MNNG), results in production of a plethora of different types of DNA lesions. In double-stranded DNA both MMS and MNNG generate mainly *N*-methylpurines: *N*^7^-methylguanine (7meG; 82% and 67% induced by MMS and MNNG, respectively) and *N*^3^-methyladenine (3meA; 11% and 12%) [1]. Although 7meG is relatively harmless, 3meA and MMS-induced *N*^3^-methylguanine (3meG; <1% [1]) are minor-groove lesions that block DNA synthesis [2–4]. In single-stranded DNA (ssDNA) and RNA MMS targets *N*^1^-adenine and *N*^3^-cytosine, subsequently producing substantial amounts of toxic *N*^1^-methyladenine (1meA) and *N*^3^-methylcytosine (3meC) lesions [5–7]. In contrast to MMS, the toxicity of MNNG also results from *O*-methylation. Under certain circumstances up to 7% of highly mutagenic MNNG-induced *O*^6^-methylguanine (O^6^meG) lesions account for the toxicity of the chemical, compared to only 0.3% generated by MMS [1,8–11].

In bacteria, as well as in eukaryotes, numerous DNA repair pathways, such as nucleotide excision repair, mismatch repair, homologous recombination and non-homologous end joining are all involved in protection of cells against the cytotoxic and mutagenic effect of alkylation damage as well as secondary DNA lesions that arise from the primary alkylation products [11,12]. However, direct damage reversal by methyltransferases and oxidative demethylases, and alkylated damage removal by base excision repair (BER) are the primary DNA alkylation repair mechanisms [5,8,11]. Oxidative demethylases (in *Escherichia coli alkB-*encoded AlkB) fix cytotoxic 1meA and 3meC residues by catalysing the hydroxylation of the methyl group [13–16]. The repair of *O*-methylation is mediated by methyltransferases (in *E*. *coli* damage-inducible Ada and constitutively expressed Ogt) by transferring the methyl groups from the lesion onto its own cysteine residues [17–20]. In addition, BER pathway repairs the majority of *N*-methylpurines, including toxic 3meA and 3meG lesions. Damage-specific DNA glycosylases recognize alkylated bases, catalyse hydrolysis of the *N*-glycosidic bond, leaving an abasic site (AP) in DNA, and subsequently initiate BER [21]. In *E*. *coli* two DNA glycosylases excise alkylation-damaged bases from DNA [22]: constitutively expressed DNA glycosylase I (*tag-*encoded Tag), specific to 3meA and 3meG [4], and damage-inducible DNA glycosylase II (*alkA-*encoded AlkA) that additionally removes 7meG and a wide variety of other substrates [23].

DNA alkylation damage can be also tolerated through a process of translesion DNA synthesis (TLS), triggered by methyl damage as a part of the SOS system [2,24,25]. TLS is mediated by specialized low-fidelity DNA polymerases that are able to catalyse past replication-blocking DNA lesions [26–29]. This damage tolerance mechanism allows to complete DNA replication in the presence of DNA damage, thereby preventing replication fork collapse and subsequent cell death [30,31]. The involvement of TLS polymerases in the bypass of cytotoxic alkylated DNA lesions reported in different organisms demonstrates the relevance of this tolerance system upon DNA alkylation damage [32–36]. For example, in *E*. *coli* the activity of TLS polymerase IV (Pol IV or DinB) was shown to be critical for survival in the presence of alkylating agents such as MMS, MNNG and ethyl methanesulfonate (EMS) [37,38]. Another *E*. *coli* Y-family SOS-activated DNA polymerase Pol V (also known as UmuD´_2_C, comprised of UmuC and UmuD proteins) is involved in error-prone TLS past 1meA/3meC lesions and AP sites, and is responsible for the MMS-induced mutagenesis [39–41].

Nevertheless, the well-characterized Pol V system is not present in all bacterial phyla. Instead, SOS-regulated *imuA-imuB-dnaE2* operon [42], renamed later as *imuA-imuB-imuC*, or *imuABC* [43], was found to contribute to the TLS in many bacterial species. This cassette encodes an *in silico* predicted SulA/RecA like DNA-binding protein ImuA, a nonfunctional homolog of Y-family DNA polymerases ImuB and an error-prone TLS DNA polymerase DnaE2 (later ImuC) [42–45]. ImuB lacks catalytic residues essential for polymerase activities, but possesses β-clamp-binding motif. Therefore, the study in *Mycobacterium tuberculosis* suggested that ImuB mediates the access of the cassette components to the replication fork through interaction with the β-clamp [46]. Furthermore, ImuA and ImuB in *M*. *tuberculosis* were shown to be essential for TLS activity of ImuC and for ultraviolet (UV)-induced mutagenesis [46,47]. In *Caulobacter crescentus imuABC* accounted for most of the UV- and mitomycin C (MMC)-induced mutations [45], but *imuC* (*dnaE2*) was not required for UV tolerance and mutagenesis in *Streptomyces coelicolor* [48]. However, little is known about the role and function of ImuABC in pseudomonads which represent one of the largest groups of bacteria including both pathogenic and non-pathogenic species. Similarly to *M*. *tuberculosis* and *C*. *crescentus*, UV-induced mutagenesis in human opportunistic pathogen *Pseudomonas aeruginosa* was shown to be ImuC-dependent [49]. In contrast, we have previously demonstrated that in soil bacterium *P*. *putida* ImuC acted as an anti-mutator in UV-irradiation experiments [50] and the involvement of ImuC in DNA replication upon UV-exposure was demonstrated only in *P*. *putida* cells lacking DNA polymerase I functions [51]. Besides, in contrast to *P*. *aeruginosa*, *P*. *putida imuA-imuB-imuC* genes are co-transcribed with the second copy of *lexA* gene, *lexA2*, which mediates the DNA damage-induced regulation of the whole operon. In *P*. *aeruginosa*, however, there is only one copy of *lexA* that regulates the *imuA-imuB-imuC* operon expression [42]. Hence, controversies surrounding ImuC function and differences in the operon organization and regulation have raised a question, whether the role of ImuC/DnaE2 in DNA damage tolerance mechanisms could vary between these two *Pseudomonas* species. Moreover, taking into account the prevalence of *imuABC* genes in a number of bacterial species, which do not pose the *umuDC* operon [44], we hypothesized that the ImuABC TLS system might have similar biological function to Pol V in response to alkylation damage. Thereby, in the present study we investigated the role of ImuC as well as the TLS DNA polymerase DinB [49,51–53] in the DNA alkylation damage tolerance and mutagenesis in two pseudomonas species, i.e. *P*. *aeruginosa* and *P*. *putida*. Unexpectedly, our findings revealed the importance of incubation temperature as a critical factor affecting the contribution of TLS polymerases in pseudomonads to the DNA alkylation damage response *in vivo*.

## Material and Methods

### Bacterial strains, plasmids and media

The bacterial strains and plasmids used in this study are listed in S1 Table. All *Pseudomonas putida* strains are derivatives of PaW85 [54], which is isogenic to KT2440 [55]. *P*. *aeruginosa* PAO1 subline PAO1-L, stored originally at the University of Lausanne, Switzerland (Dieter Haas collection) was obtained in 2013 from AP Stephan Heeb, University of Nottingham, United Kingdom. *P*. *aeruginosa* and *E*. *coli* were grown in Luria-Bertani (LB) medium [56], *P*. *putida* was grown in LB or in M9 medium [57] supplemented with casamino acids (CAA) and glucose at final concentrations of 0.4% and 0.2%, respectively. Minimal solid medium was M9 supplemented with glucose and contained 1.5% Difco agar. LB solid medium for Rif^r^ mutation assay contained 1.5% Difco agar and rifampicin at 100 μg/ml. Other antibiotics were added at following concentrations: ampicillin at 100 μg/ml, kanamycin at 50–500 μg/ml, streptomycin at 200 μg/ml, benzylpenicillin at 1500 μg/ml and carbenicillin at 200–500 μg/ml. Bacteria were incubated on agar plates or in liquid cultures with shaking (180 rpm): *P*. *aeruginosa* and *E*. *coli* at 37°C and *P*. *putida* at 30°C, if not specified otherwise. *P*. *aeruginosa*, *P*. *putida* and *E*. *coli* were electrotransformed as described by [58]. *E*. *coli* strains DH5α (Invitrogen, USA), DH5α λpir [59] and CC118 λpir [60] were used for DNA cloning procedures, and HB101 [61] was used as a host for helper plasmid pRK2013 [62], which was necessary for the mobilization of non-conjugative plasmids.

### Cloning and construction of strains

For the construction of deletion mutants and suicide pEMG plasmids listed in S1 Table, we used the protocols of Martínez-García and de Lorenzo [59]. Briefly, for the deletion of *P*. *putida imuA* (PP3117), *imuB* (PP3118), *imuC* (PP3119), *imuABC* (PP3117-PP3119), *dinB* (PP1203), *tag* (PP0062) and *P*. *aeruginosa imuA* (PA0671), *imuB* (PA0670), *imuC* (PA0669), *imuABC* (PA0671-PA0669), *dinB* (PA0923) and *alkA* (PA1686) genes the corresponding recombinogenic plasmids were generated (S1 Table). For that, the upstream and downstream regions (600–1000 bp) of the gene to be deleted were first amplified separately with two primer pairs (Ts1 and Ts2) (S2 Table) and then joined into one fragment by overlap extension PCR. The obtained DNA fragments were digested with the BamHI, XbaI, SacI, or Acc65I (S2 Table) and ligated into the corresponding sites of the pEMG plasmid. Subsequently, the obtained plasmids were conjugatively transferred into the *P*. *putida* strain PaW85 and *P*. *aeruginosa* PAO1 subline PAO1-L by using the helper plasmid pRK2013. *P*. *putida* and *P*. *aeruginosa* transconjugants carrying a cointegrate in the chromosome were isolated on kanamycin selective plates, then electrotransformed with the I-SceI expression plasmid pSW(I-SceI) and selected on benzylpenicillin or carbenicillin containing plates, respectively. Kanamycin-sensitive colonies were selected, and the deletion of the corresponding genes was verified by PCR. Plasmid pSW(I-SceI) was eliminated from the deletion strains by growing bacteria in LB medium overnight without antibiotics.

For the complementation of *P*. *putida imuB*-deficient strain, the overexpression cassette for the *imuB* gene was inserted into *P*. *putida* chromosome within the miniTn*7* transposon. For that, the suicide vector pBK-miniTn*7*-ImuB was constructed. First, the 1471-kb DNA fragment containing the *imuB* gene was amplified from the chromosome of *P*. *putida* PaW85 by using primers dinb2lookus and dinb2alamus (S2 Table) and inserted into the SmaI-cleaved pUCNotlacItac, creating the plasmid pUCNotlacItac-ImuB. The plasmid pUCNotlacItac-ImuB was cut with NdeI and the ends of DNA were blunted with Klenow fragment. This was followed by the digestion with KpnI to obtain the DNA fragment containing the *lacI*^q^*-P*_*tac*_-*imuB* expression cassette. The *lacI*^q^*-P*_*tac*_-*imuB* cassette was inserted into the KpnI and SmaI sites of pBK-miniTn*7*-ΩSm1 [63]. The constructed suicide vector pBK-miniTn*7*-ImuB was integrated into the *P*. *putida* Δ*imuBalkA* strain by electroporation as described [64], creating the strain PpΔ*imuBalkA+B* (S1 Table).

For the construction of pJB-PadinB expression plasmid (S1 Table), *dinB* gene was amplified from the *P*. *aeruginosa* chromosome by using PAO_dinB_RBS and PAO_dinBalu_BglII primers (S2 Table). The 1086-bp PCR fragment was cut with XbaI and BglII and inserted into pJB-*lacI*^q^-P_*tac*_ vector, digested with the same restriction enzymes, to obtain pJB-PadinB.

For the construction of transcriptional fusion of the *dinB* promoter with *lacZ* reporter, the promoter region of *dinB* gene was amplified from the *P*. *aeruginosa* chromosome by using primers PaoDinB_prom_F_BamH and PaoDinB_prom_R_BamH (S2 Table) and cloned into BamHI site of the pBLKT plasmid to obtain pBLKT-PAdinB (S1 Table).

For the construction of p9TT_B_lacZ-lexA2 (S1 Table) transcriptional fusion, the *lexA2* promoter region from the *P*. *putida* chromosome was amplified by using primers LexA2-140BamH and LexA2+92BamH (S2 Table), complementary to the upstream and downstream regions of the *lexA2* promoter, respectively. The 272-bp product was cleaved with BamHI and inserted into p9TT_B_lacZ to obtain the plasmid p9TT_B_lacZ-lexA2.

### Analysis of MMS and MNNG sensitivity and mutagenesis studies

Several independently isolated clones for each genotype were used in assays monitoring DNA alkylation tolerance and in mutagenesis studies to examine reproducibility of the results. To estimate the MMS and MNNG sensitivity of bacteria, the overnight cultures of *P*. *aeruginosa* and *P*. *putida* grown in liquid LB medium were serially diluted into M9 medium and 5 μl aliquots of 10-fold serial dilutions of cultures were spotted onto LB plates and LB plates containing the indicated concentrations of MMS and MNNG. Sensitivity of strains was estimated by counting colony-forming units per ml (CFU/ml) after 24 or 48 h of incubation of plates in the dark at 37°C or 30°C. At least four independent experiments were performed in triplicate.

MMS-induced liquid killing was used to estimate the MMS-sensitivity of *P*. *putida* exponentially growing TLS-deficient cells. For that, overnight cultures were diluted 1:100 into fresh LB medium and incubated for 3 hours with agitation at 30°C. At this point, cultures were split in triplicates and treated with 25 mM and 30 mM MMS for 45 min at 30°C with shaking. Afterwards, cells were harvested by centrifugation, washed twice in M9 medium, suspended in the same volume of M9 medium and 10-fold dilutions of the cultures were spotted on LB plates. The survivors of the MMS treatment were determined by colony count per ml (CFU/ml) after overnight incubation at 30°C. Three independent experiments were performed in triplicate.

To estimate the effect of incubation temperature on the survival of MMS-challenged *P*. *aeruginosa* ImuC*-*proficient and -deficient Δ*alkA* strains, overnight cultures were diluted 1:100 into fresh LB medium and grown aerobically at 37°C for 3 hours. Next, cultures were challenged with 2.5 mM MMS for 45 min at 37°C, harvested by centrifugation, washed twice in M9 medium, suspended in the same volume of M9 medium and 10-fold dilutions of the cultures were spotted in parallel on LB plates and incubated at 37°C and 30°C overnight. At least four independent experiments were performed in triplicate.

To estimate the frequency of mutations induced by MMS, overnight cultures of *P*. *putida* and *P*. *aeruginosa* were diluted 1:100 into fresh glcCAA and LB medium, respectively, and incubated for 3.5 hours, until the OD_580_ reached 1.0 to 1.2. Thereafter, 40 μl of mid-exponential *P*. *putida* cell culture was inoculated into 2 ml of fresh glcCAA medium and 100 μl of mid-exponential *P*. *aeruginosa* culture into 5 ml of fresh LB medium containing the indicated concentrations of MMS (0.15 mM or 0.05 mM) and cultured overnight with aeration. Overnight cultures were plated onto LB plates containing 100 μg/ml rifampicin and incubated for 48 h. To count the total number of viable cells in the overnight cultures, appropriate dilutions of the same cultures were spotted onto LB plates. Following the counts, the frequencies of Rif^R^ mutations (number of Rif^R^ mutants per plated 10^9^ cells) were calculated. Data presented represents mean (±SE) values from 7–10 replicates in 3–5 independent experiments.

### Measurement of β-galactosidase activity

To assess DNA damage induced by MMS treatment, *we studied P*. *putida lexA2 promoter* activity [42] by using the β-galactosidase assay [56]. The level of expression of β-galactosidase was monitored on the plasmid p9TT_B_lacZ-lexA2 carrying the *lexA2* promoter fused with the *lacZ* reporter gene. The overnight cultures of *P*. *putida* strains harboring p9TT_B_lacZ-lexA2 fusion were diluted 1:50 into 25 ml of fresh LB medium and incubated at 30°C with shaking for 3 hours (OD_580_ 1.0). At this point, the cultures were divided into individual test tubes of 2 ml each and 0.3 mM MMS was added. Next, the test tubes were aerated at 30°C or 37°C. Samples for the β-galactosidase assay were taken from the cultures grown for 1 h and 22 hrs, and β-galactosidase activity was measured as described previously [56]. Five independent experiments were performed in triplicate.

To assess the promoter activity of *P*. *aeruginosa dinB* gene at different temperatures, we monitored the level of expression of β-galactosidase by using the plasmid pBLKT-PAdinB carrying the *dinB* promoter fused with the *lacZ* reporter gene. The overnight cultures of *P*. *aeruginosa* strains harboring pBLKT-PAdinB fusion were diluted 1:50 into 50 ml of fresh LB medium and incubated at 37°C with shaking until OD_580_ reached 0.8. At this point, the cultures were divided into individual test tubes of 2 ml each and incubated with 0.5 mM MMS with aeration at 30°C or 37°C for 18 hrs, and β-galactosidase activity was measured as described previously [56]. Five independent experiments were performed in quintuplicate.

### Statistical analysis

Data without normal distribution was analyzed using the Kruskal-Wallis test, followed by the Dunn’s post hoc test to investigate the differences among groups. ANOVA followed by Tukey's or Bonferroni’s post hoc test was used to evaluate the differences when two or more factors were analysed. For all statistical tests the significance level was set at *P* < 0.05. The calculations were performed using Statistica 13 software or GraphPad Prism version 6.00 for Windows (GraphPad Software, La Jolla California USA, www.graphpad.com). Graphs were constructed using GraphPad Prism version 6.00.

## Results

### TLS contributes differently to survival of the wild-type *P*. *putida* and *P*. *aeruginosa* upon alkylation damage

**Fig 1:**
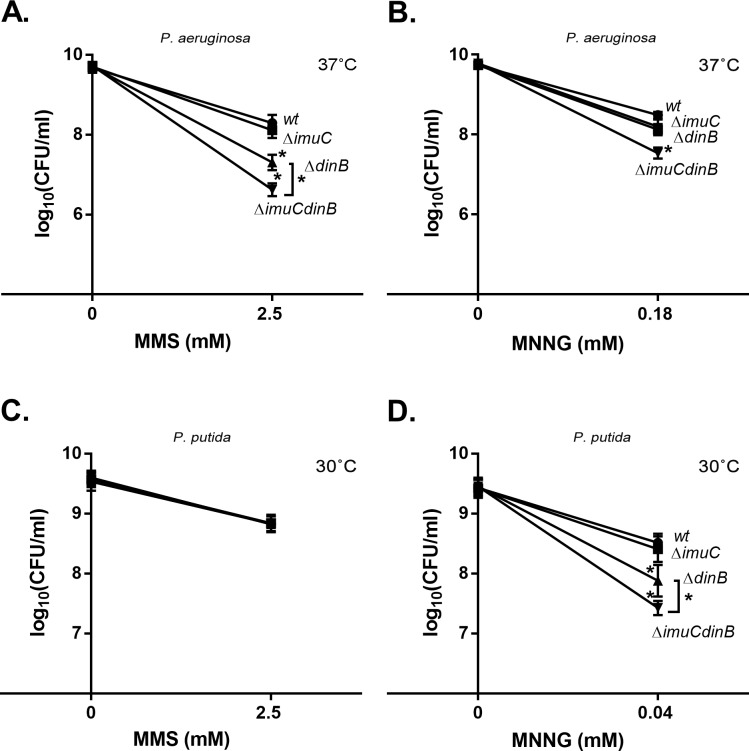
Sensitivity of *P*. *aeruginosa* and *P*. *putida* wild-type and their TLS polymerase-deficient derivatives to MMS and MNNG. Sensitivity was estimated by spotting 10-fold dilutions of overnight cultures of *P*. *aeruginosa* (A, B) and *P*. *putida* (C, D) onto LB plates containing MMS (A, C) or MNNG (B, D). *P*. *aeruginosa* was incubated at 37°C for 24 h (A, B). *P*. *putida* was incubated at 30°C on MMS-containing plates for 48 h (C) and on MNNG for 24 h (D). Data represents the mean (±95%CI) values. (●) wild-type; (■) Δ*imuC*; (▲) Δ*dinB*; (▼) Δ*imuCdinB*. Asterisks indicate statistically significant difference (maximum value *P* < 0.05; two-way ANOVA followed by Tukey’s multiple comparison post hoc test) in sensitivity between the mutant and the wild-type strain or between the Δ*dinB* and the Δ*imuCdinB* (A, D).

In order to investigate the implication of specialized DNA polymerases in alkylation damage tolerance in pseudomonads, we constructed a set of *imuC* and *dinB* deletion derivatives of *P*. *putida* PaW85 and *P*. *aeruginosa* PAO1 (PAO1-L subline) and examined the sensitivity of these strains to the DNA alkylating agents by performing a serial dilution drop test. We observed that the deletion of *dinB* in *P*. *aeruginosa* sensitized cells to MMS treatment (Fig 1A; *P* ≤ 0.0001), denoting an essential role of DinB in the protection against MMS-induced damage. Meanwhile, MMS did not affect the survival of ImuC-deficient *P*. *aeruginosa* strain. This result is consistent with previous observation that *P*. *aeruginosa* PAO1 mutant lacking the *imuABC* operon has no increased sensitivity to MMS-mediated damage [53]. However, we observed that *P*. *aeruginosa* strain deficient for both *dinB* and *imuC* displayed significantly increased MMS sensitivity in comparison with *P*. *aeruginosa* lacking only DinB (Fig 1A; *P* ≤ 0.0001). These results suggest that ImuC is also implicated in cellular tolerance to MMS as a backup polymerase, being crucial for survival in the absence of DinB upon exposure to alkylating agents.

Different from *P*. *aeruginosa*, neither the deletion of *dinB* or *imuC*, nor the deletion of both genes in *P*. *putida* affected the survival of bacteria on MMS-containing plates (Fig 1C). This defines a distinct contribution of TLS in these organisms to the protection of wild-type cells against MMS-induced damage. Nevertheless, *imuC* and *dinB* double deficient *P*. *aeruginosa* and *P*. *putida* strains both displayed reduced survival in the presence of MNNG (Fig 1B and 1D; *P* ≤ 0.0001). Moreover, *P*. *putida* Δ*dinB* strain also displayed slightly increased MNNG sensitivity, whereas the sensitivity of the *imuCdinB* double mutant was significantly higher than that of the Δ*dinB* strain (Fig 1D, *P* ≤ 0.0001). Since MNNG also targets oxygen atoms in the DNA [1], the increased susceptibility of the Δ*imuCdinB* bacteria to MNNG implied a possible involvement of DinB and ImuC in the bypass or repair of *O*-alkylation damage in these organisms.

**Fig 2:**
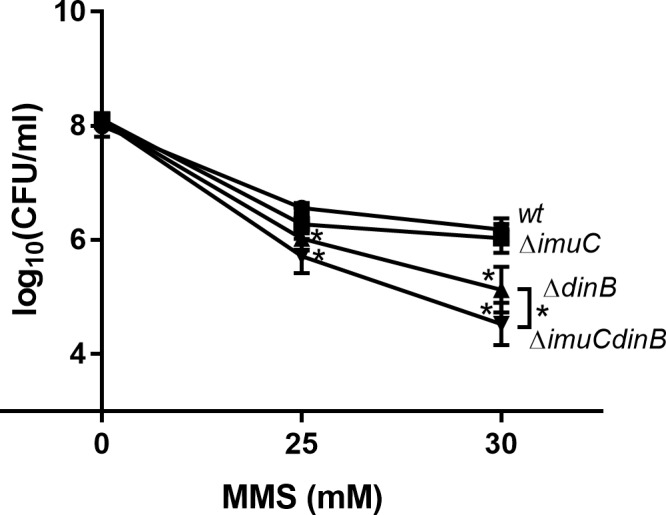
Survival of exponentially growing *P*. *putida* TLS polymerase-deficient cells after MMS treatment. Survival was estimated at different concentrations of MMS after 45-min treatment period. Data represents the mean (±95%CI) values of three independent experiments performed in triplicate. (●) wild-type; (■) Δ*imuC*; (▲) Δ*dinB*; (▼) Δ*imuCdinB*. Asterisks indicate statistically significant difference (*P* < 0.05; two-way ANOVA followed by Tukey’s multiple comparison post hoc test) in sensitivity between the mutant and the wild-type strain, and between the Δ*dinB* and the Δ*imuCdinB* strains.

It is important to note that when *P*. *putida* cells were incubated on the MMS-containing plates, the growth of bacteria was inhibited, and the colonies appeared only after 2 days of incubation. This observation led us to conduct a killing experiment in a liquid medium. Analysis of *P*. *putida* TLS-deficient strains exposed to MMS during exponential growth phase revealed the negative effect of TLS-deficiency on the survival of bacteria (Fig 2). Specifically, the survival of exponentially growing DinB-deficient strain following MMS challenge in liquid medium was slightly lower than the survival of wild-type bacteria (*P* ≤ 0.05 at 25 mM MMS and *P* ≤ 0.0001 at 30 mM MMS). In addition, the *imuCdinB* double-deficient strain was significantly more sensitive to the killing with 30 mM MMS than the *dinB*-deficient mutant (*P* ≤ 0.01). Thus, these results point at the increased requirement of both TLS polymerases for the survival of rapidly replicating *P*. *putida* cells in the presence of MMS and demonstrate that ImuC and DinB are involved in the DNA alkylation damage tolerance in both *P*. *putida* and *P*. *aeruginosa*.

### ImuC and DinB are essential for the survival of bacteria with impaired glycosylase-mediated DNA repair upon MMS- and MNNG-induced damage

In *E*. *coli* the MMS-induced DNA replication-blocking 3meA and 3meG lesions are removed by DNA glycosylases Tag and AlkA as a part of the BER pathway [4,65]. In addition, the DNA glycosylase AlkA in *P*. *putida* is thought to be involved in the repair of toxic 1meA and 3meC [66]. Thus, we constructed *P*. *putida* TLS-deficient strains defective additionally in DNA glycosylase-mediated DNA repair, lacking either the DNA glycosylase II (*alkA*) only or both enzymes (*alkAtag*), and *P*. *aeruginosa* TLS-deficient strains, lacking *alkA*, to specify the role for TLS in cellular tolerance to *N*-alkyl lesions that accumulate in cells because of BER imbalances.

**Fig 3:**
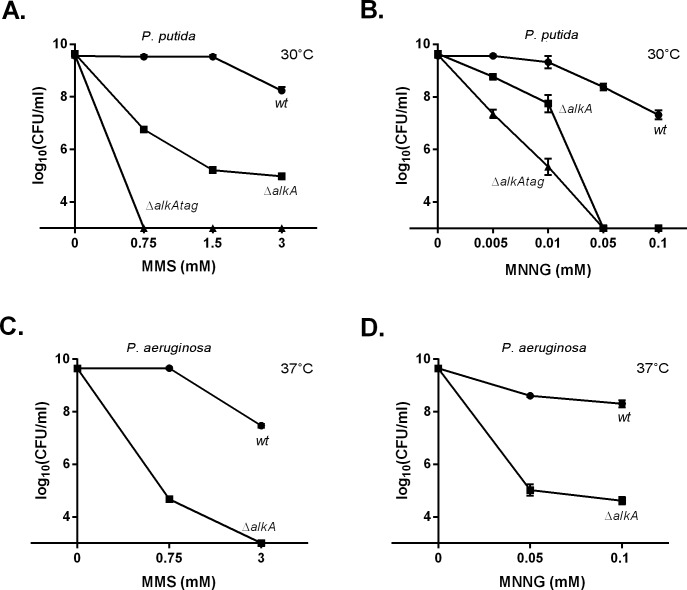
Sensitivity of *P*. *putida* and *P*. *aeruginosa* wild-type and their DNA glycosylase-deficient derivatives to MMS and MNNG. Sensitivity was estimated by spotting 10-fold dilutions of overnight cultures of *P*. *putida* (A, B) and *P*. *aeruginosa* (C, D) onto LB plates containing different concentrations of MMS (A, C) and MNNG (B, D). Data represents the mean (±95%CI) values. (●) wild-type; (■) Δ*alkA*; (▲) Δ*alkAtag*. *P*. *putida* was incubated at 30°C and *P*. *aeruginosa* was incubated at 37°C.

**Fig 4:**
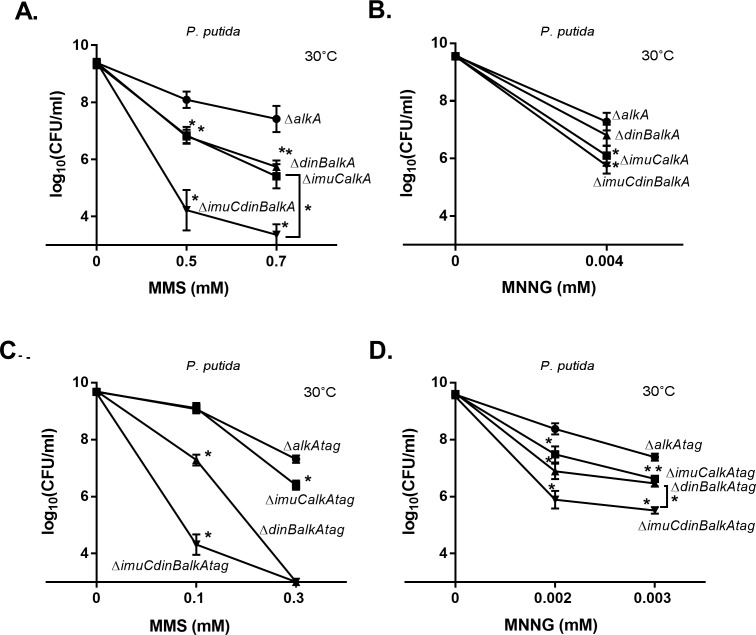
Sensitivity of *P*. *putida* Δ*alkA* and Δ*alkAtag* strains and their different TLS polymerase-deficient derivatives to MMS and MNNG. Sensitivity was estimated by spotting 10-fold dilutions of overnight cultures of *P*. *putida* Δ*alkA* (A, B) and Δ*alkAtag* (C, D) strains onto LB plates containing different concentrations MMS (A, C) and MNNG (B, D) and incubated at 30°C for 24 h. Data represents the mean (±95%CI) values. (●) Δ*alkA*; (■) Δ*imuCalkA*; (▲) Δ*dinBalkA*; (▼) Δ*imuCdinBalkA* (A, B); (●) Δ*alkAtag*; (■) Δ*imuCalkAtag*; (▲) Δ*dinBalkAtag*; (▼) Δ*imuCdinBalkAtag* (C, D). Asterisks indicate statistically significant difference (*P* < 0.05; two-way ANOVA followed by Tukey’s multiple comparison post hoc test) in the sensitivity of the Δ*alkA* or the Δ*alkAtag* mutant in comparison with the other deletion mutants. In addition, significant difference in sensitivity between the Δ*dinBalkA* or Δ*imuCalkA* and the Δ*imuCdinBalkA* (A); Δ*dinBalkAtag* or Δ*imuCalkAtag* and the Δ*imuCdinBalkAtag* strains (D) is indicated.

**Fig 5:**
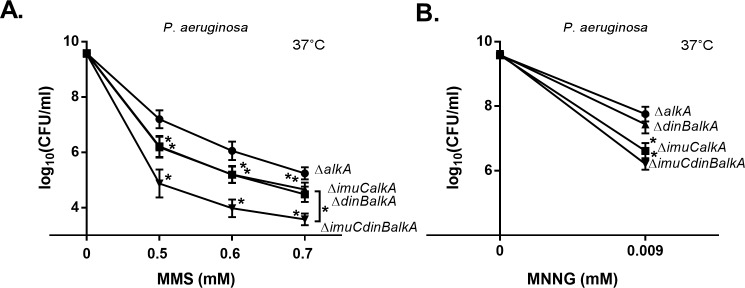
Sensitivity of *P*. *aeruginosa* Δ*alkA* strain and its TLS polymerase-deficient derivatives to MMS and MNNG. Sensitivity was estimated by spotting 10-fold dilutions of overnight cultures onto LB plates containing different concentrations of MMS (A) and MNNG (B), and incubated at 37°C for 24 h. Data represents the mean (±95%CI) values. (●) Δ*alkA*; (■) Δ*imuCalkA*; (▲) Δ*dinBalkA*; (▼) Δ*imuCdinBalkA*. Asterisks indicate statistically significant difference (*P* < 0.05; two-way ANOVA followed by Tukey’s multiple comparison post hoc test) in the sensitivity of the Δ*alkA* mutant in comparison with the other deletion mutants. In addition, significant difference in sensitivity between the strains Δ*imuCalkA* or Δ*dinBalkA* and the Δ*imuCdinBalkA* (A) is indicated.

The hypersensitivity of *P*. *putida* strains deficient in the DNA glycosylases AlkA and Tag (Fig 3A and 3B) and *P*. *aeruginosa* strain deficient in AlkA (Fig 3C and 3D) upon MMS and MNNG exposure emphasized the primary role of BER in the protection of wild-type bacteria against simple alkylating agents. Subsequent characterization of glycosylase deficient mutants enabled us to further elucidate the contribution of DinB and ImuC to DNA alkylation damage tolerance (Figs 4 and 5). Namely, *P*. *putida* Δ*imuCalkA* and Δ*dinBalkA* mutants displayed an increased sensitivity to MMS in comparison with Δ*alkA* cells (Fig 4A; *P* ≤ 0.0001). This demonstrates that both ImuC and DinB are required for the survival of the *alkA-*deficient *P*. *putida* upon MMS exposure, suggesting their involvement in replication past lesions normally repaired by AlkA. Moreover, the Δ*imuCdinBalkA* strain was significantly more sensitive to MMS than the Δ*imuCalkA* and Δ*dinBalkA* mutants (*P* ≤ 0.0001), implying that the specificity of ImuC and DinB in alkylation lesion bypass might be different. In the response to MNNG, however, the survival of the AlkA-deficient *P*. *putida* was negatively affected only by the absence of ImuC (Fig 4B; *P* ≤ 0.0001). In addition, the sensitivity of the Δ*imuCdinBalkA* triple mutant was comparable to that of the Δ*imuCalkA* double mutant, demonstrating that the survival of the AlkA*-*deficient bacteria upon MNNG treatment in *P*. *putida* is mostly ImuC-dependent. Different from the Δ*alkA* strain, the importance of DinB for the survival of the Δ*alkAtag* bacteria following alkylating treatment was markedly higher, as Δ*dinBalkAtag* strain was significantly more sensitive to MMS than the Δ*imuCalkAtag* cells (Fig 4C). However, the fact that the Δ*alkAtag* cells deficient in both DinB and ImuC were hypersensitive to MMS and MNNG (Fig 4C and 4D) corroborated the hypothesis that DinB and ImuC can bypass distinct DNA replication-blocking lesions.

The survival phenotype of the *P*. *aeruginosa* TLS-deficient Δ*alkA* strains was similar to the DNA alkylation damage sensitivity profile of the corresponding *P*. *putida* mutants (Fig 4A and 4B and Fig 5A and 5B). The deletion of either DinB or ImuC in the Δ*alkA* bacteria resulted in the increased MMS sensitivity (*P* ≤ 0.0001), and the Δ*imuCdinBalkA* strain was significantly more sensitive to MMS than the Δ*imuCalkA* and Δ*dinBalkA* mutants (Fig 5A). In addition, similar to *P*. *putida*, ImuC in *P*. *aeruginosa* appeared to be more critical than DinB for the protection of the *alkA-*deficient bacteria against the MNNG-mediated damage (Fig 5B). Taken together, these results imply that ImuC and DinB facilitate the survival of AlkA-deficient bacteria by carrying out TLS possibly past similar DNA alkylation lesions in both *Pseudomonas* species.

### Both ImuA and ImuB are required for the functionality of ImuC

**Fig 6:**
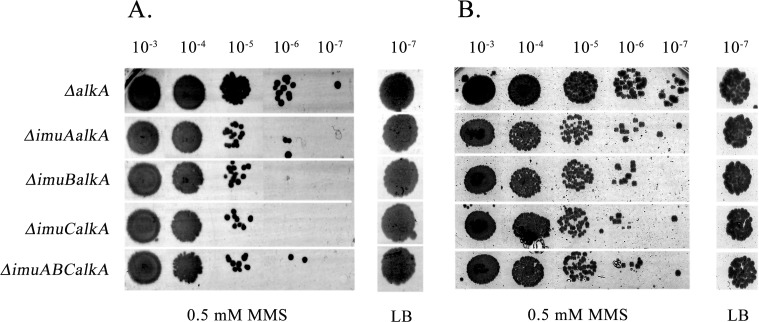
Study of the requirement of *imuA* and *imuB* for the alkyl damage tolerance. **S**ensitivity of *P*. *aeruginosa* (A) *and P*. *putida* (B) Δ*alkA* strains and their *imuA-*, *imuB-*, *imuC-* and *imuABC*-deficient derivatives to MMS was compared. Sensitivity was estimated by spotting 10-fold dilutions of overnight cultures of *P*. *aeruginosa* or *P*. *putida* onto LB plates supplemented with 0.5 mM MMS and incubated at 37°C or 30°C for 24 h, respectively.

Studies in *M*. *tuberculosis* and *C*. *crescentus* have demonstrated that all products of the *imuABC* gene cassette are essential for ImuC to function as a TLS polymerase [45,46]. Therefore, we next investigated whether the absence of *imuA* or *imuB* affects the alkyl damage tolerance in pseudomonads. The deletion of either *imuA* or *imuB*, like the deletion of *imuC*, sensitized *P*. *aeruginosa* and *P*. *putida* AlkA-deficient strains to the alkylating agents (Fig 6A and 6B), and the detected effects were comparable to that of the Δ*imuABCalkA* mutant. In addition, the increased sensitivity of the ImuB-deficient Δ*alkA* strain to the DNA alkylation damage was not associated with a possible polar effect of the *imuB* gene deletion on the *imuC* gene expression. Namely, the introduction of an IPTG (isopropyl-β-D-thiogalactopyranoside)-inducible *lacItacimuB* gene cassette into the *att*Tn*7* site of the genome of the ImuB-deficient *P*. *putida* Δ*alkA* cells almost completely restored the phenotype to the parental Δ*alkA* strain level (S1 Fig). Thus, our data indicate that ImuB and ImuA are both required for the involvement of ImuC in the DNA alkyl damage tolerance in pseudomonads.

### ImuC is responsible for the MMS-induced mutability in *P*. *putida* and *P*. *aeruginosa* cells

As MMS-induced mutagenesis in *E*. *coli* is associated with the error-prone activity of Pol V [39,67], we asked next whether the ImuC- or DinB-mediated TLS contributes to MMS mutability in pseudomonas species. The effects of the *imuC* and *dinB* deletions on MMS-induced mutagenesis were examined in bacteria incubated with low concentrations (commonly 0.15 mM) of MMS overnight. Since the survival of *P*. *putida* Δ*dinBalkAtag* and Δ*imuCdinBalkAtag* strains at this concentration was too low, we then monitored the effect of TLS polymerases on the MMS-induced mutagenesis in the AlkATag-deficient bacteria in the presence of 0.05 mM MMS.

**Fig 7:**
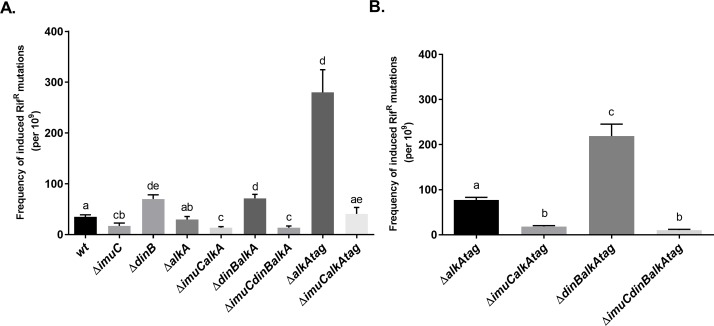
Effect of TLS polymerase deficiencies on the frequencies of MMS-induced Rif^R^ mutations in *P*. *putida* wild type and *alkA*- and *alkAtag*-deficient backgrounds. Bacteria were exposed to 0.15 mM MMS (A) or to 0.05 mM MMS (B) overnight. Data represents the mean (±SE). Letters indicate homogeneous groups. Groups that have no common letter are significantly different at *P* < 0.05, according to Kruskal-Wallis test followed by Dunn's multiple comparisons test (e.g., group with the letter ‘a’ is significantly different from the group with the letters ‘cb’, but not from the groups with the letters ‘ab’ or ‘ae’).

The frequency of MMS-induced Rif^R^ mutations in the AlkA-deficient *P*. *putida* strain was comparable to that measured in the wild-type strain (Fig 7A). However, the MMS-induced Rif^R^ mutant frequency in the AlkATag-deficient bacteria was significantly higher compared to the Δ*alkA* and wild-type strains (Fig 7A, *P* ≤ 0.0001). These results indicated that the DNA glycosylases-mediated repair is important for the suppression of mutagenic effects of MMS. Moreover, in the presence of 0.15 mM MMS the number of MMS-induced Rif^R^ mutants in the Δ*alkAtag* bacteria was remarkably (3.6 fold) higher than when incubated with 0.05 mM (Fig 7A and 7B), demonstrating a strong correlation between the concentration of MMS and the frequency of induced mutations. More importantly, MMS-induced mutant frequency in the *alkA-* and *alkAtag-*proficient and *-*deficient bacteria lacking ImuC was significantly lower compared to the corresponding ImuC-proficient strains, indicating that ImuC is responsible for the MMS mutability in *P*. *putida* (Fig 7). In contrast, the deletion of *dinB* gene in the AlkA*-* and AlkATag-deficient and -proficient bacteria resulted in the increased frequency of MMS-induced mutations (Fig 7A and 7B). The mutant frequency in the Δ*imuCdinBalkA* and Δ*imuCdinBalkAtag* strains was similar to that of the Δ*imuCalkA* and the Δ*imuCalkAtag* strains, respectively. This suggests that the mutator phenotype observed in the Δ*alkA* and Δ*alkAtag* DinB-deficient bacteria is ImuC-dependent. Moreover, our data demonstrates that DinB in *P*. *putida* is involved in the suppression of mutagenic activity of ImuC and in the accurate synthesis past MMS-induced damage.

**Fig 8:**
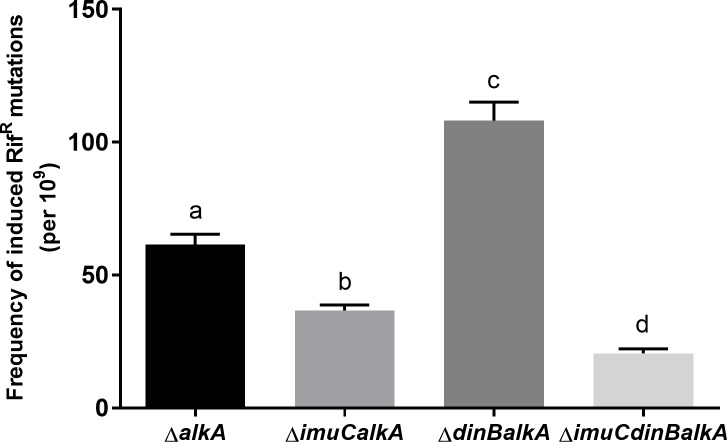
Effect of the ImuC and DinB deficiencies on the frequencies of MMS-induced Rif^R^ mutations in *P*. *aeruginosa alkA*-deficient bacteria. Bacteria were exposed to 0.15 mM MMS overnight. Data represents the mean (±SE). Groups that have no common letter are significantly different at *P* < 0.05, according to Kruskal-Wallis test followed by Dunn's multiple comparisons test.

Similarly to *P*. *putida*, MMS-induced mutagenesis in the *P*. *aeruginosa* Δ*alkA* background was largely influenced by the presence of ImuC (Fig 8). The deletion of *dinB* in *P*. *aeruginosa* also resulted in the increased number of MMS-induced mutations, pointing at the DinB-mediated suppression of ImuC-dependent MMS-induced mutagenesis. Interestingly, the frequency of MMS-induced Rif^R^ mutants in *P*. *aeruginosa* AlkA-deficient strain lacking both ImuC and DinB was lower in comparison to that when only ImuC was absent (Fig 8, *P* = 0.0115). This suggests that in the absence of TLS other DNA repair pathways mediate more accurate alkylation-damage repair.

### Alkylation damage tolerance in *P*. *aeruginosa* is temperature dependent

**Fig 9:**
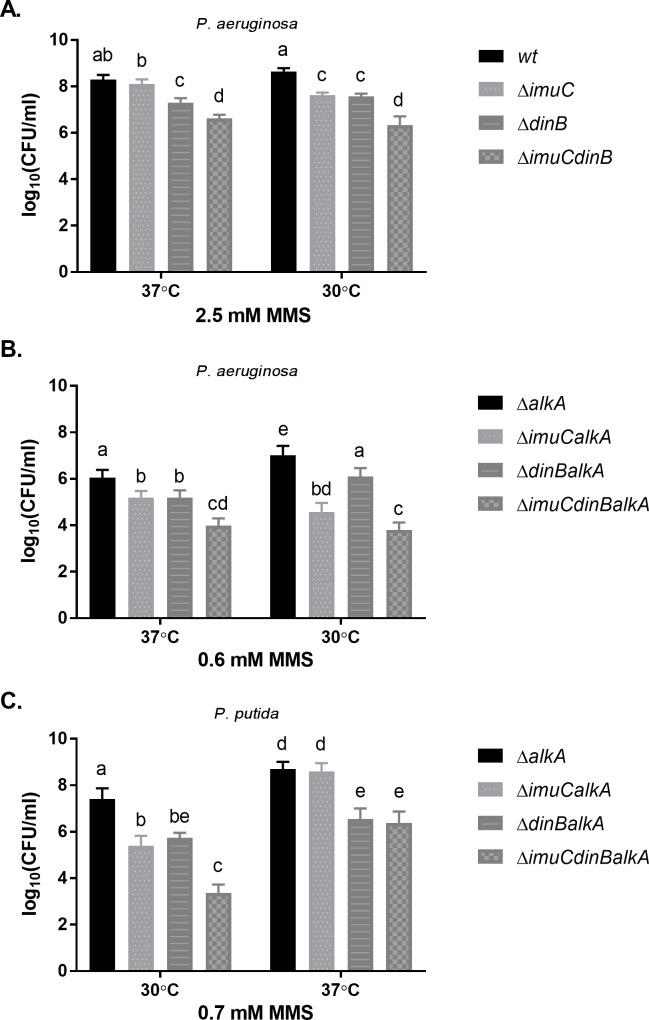
Effect of incubation temperature on the sensitivity of *P*. *aeruginosa* and *P*. *putida* TLS polymerase-deficient strains to MMS. 10-fold dilutions of overnight cultures of *P*. *aeruginosa* (A, B) and *P*. *putida* (C) were spotted in parallel onto LB plates containing different concentrations of MMS and incubated at 37°C and 30°C. Sensitivity of *P*. *aeruginosa* TLS polymerase-deficient strains to 2.5 mM MMS (A); sensitivity of *P*. *aeruginosa* TLS polymerase-deficient Δ*alkA* mutants to 0.6 mM MMS (B); sensitivity of *P*. *putida* TLS polymerase-deficient Δ*alkA* mutants to 0.7 mM MMS (C) is demonstrated. *P*. *aeruginosa* strains (A, B) were incubated at 37°C for 24 h and at 30°C for 48 h; *P*. *putida* (C) was incubated at 37°C and 30°C for 24 h. Data represents the mean (±95%CI) values. Letters indicate homogeneous groups according to ANOVA followed by Bonferroni’s multiple comparisons test (*P* < 0.05).

**Fig 10:**
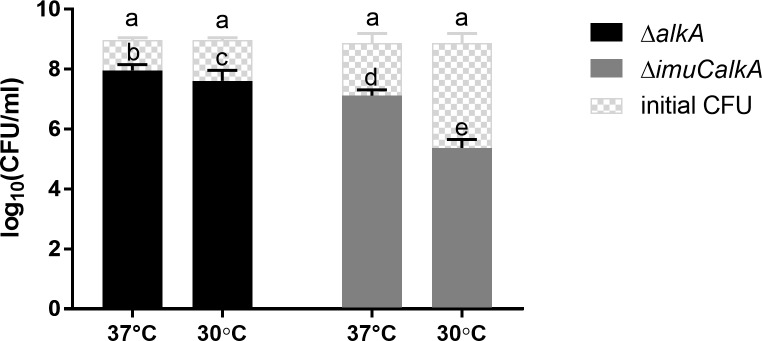
Effect of incubation temperature on the survival of MMS-treated *alkA*-deficient bacteria. The survival of *P*. *aeruginosa* Δ*alkA* (black) and Δ*imuCalkA* (grey) strains at 37°C or 30°C after treatment with 2.5 mM MMS for 45-min period is indicated. Data represents the mean (±SD) values. Columns with the pattern fill represent the initial CFU/ml. Letters indicate homogeneous groups according to ANOVA followed by Bonferroni’s multiple comparisons test (*P* < 0.05).

Surprisingly, we noticed that the growth temperature of bacteria affected the MMS-sensitivity of TLS-deficient *P*. *aeruginosa* strains. When incubated at optimum growth temperature at 37°C [68], *P*. *aeruginosa* strains deficient in *dinB* exhibited increased sensitivity to MMS compared to the wild-type (Figs 1A and 9A). However, at 30°C the loss of ImuC also led to the reduced survival of bacteria in the presence of MMS (Fig 9A). Moreover, when the AlkA-deficient mutants were incubated at 30°C, the presence of ImuC became significantly more important for the survival than the presence of DinB (Fig 9B). However, incubation of *P*. *aeruginosa* on MMS-containing plates at 30°C also significantly slowed down the growth of bacteria, as clearly visible colonies appeared only after two days of incubation. In order to exclude the effect of prolonged incubation, we performed a MMS-induced killing experiment in liquid medium and determined the survival of MMS-treated bacteria under different temperatures (30°C and 37°C). We observed a slight effect of the incubation temperature on the survival of the MMS-treated Δ*alkA* strain (Fig 10, *P* = 0.033). However, the survival of the MMS challenged Δ*imuCalkA* cells after overnight incubation at 37°C was up to 10-fold higher than those incubated at 30°C (Fig 10; *P* ≤ 0.0001). Taken together, our results demonstrate that the survival of TLS-deficient *P*. *aeruginosa* mutants depends on the growth temperature of bacteria and there is an increased requirement for ImuC in cellular protection against MMS-induced killing at 30°C.

**Fig 11:**
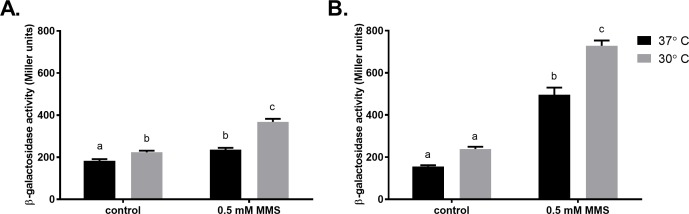
Effect of incubation temperature on transcription from the *P*. *aeruginosa dinB* promoter. β-galactosidase activity expressed from the *dinB* promoter-*lacZ* reporter was measured in the *P*. *aerugionsa* wild-type (A) and in its *alkA-*deficient derivative (B) incubated at 37°C (black) and 30°C (grey) overnight in liquid LB medium supplemented with 0.5 mM MMS or not (control). Data represents the mean (±SE) values. Letters indicate homogeneous groups according to ANOVA followed by Bonferroni’s multiple comparisons test (*P* < 0.05).

Taking into account the fact that the survival of Δ*imuCdinB* strain was quite similar at both temperatures (Fig 9A), we hypothesized that the increased sensitivity of ImuC-deficient cells at 30°C could result from the diminished ability of DinB to single-handedly protect against accumulating alkylating damage (two polymerase affair). First, we tested whether the incubation temperature affected the transcriptional activity of *dinB*. The basal promoter activity of the *dinB* gene in unstressed cells was slightly higher in *P*. *aeruginosa* incubated at 30°C (Fig 11). In MMS-treated bacteria *dinB* expression was up-regulated. However, in cells incubated at 30°C the β-galactosidase activity was significantly higher. In addition, transcription from the *dinB* promoter measured in *alkA*-deficient bacteria exposed to the same concentration of MMS was almost 2-times higher than in the wild-type at both temperatures (Fig 11B). Since *dinB* is DNA-damage inducible [49], the increased *dinB* promoter activities observed in bacteria incubated at 30°C and in cells with impaired BER functions could indicate elevated levels of DNA damage upon MMS exposure. Next, we introduced a plasmid carrying the *dinB* gene under the *P*_*tac*_ promoter into ImuC-deficient *P*. *aeruginosa* to examine whether the increased lethality of bacteria in the absence of ImuC might be associated with accumulation of DNA lesions bypassed also by DinB. Indeed, the overexpression of *dinB* increased the MMS tolerance of the Δ*imuC* strain and even the Δ*imuCdinB* strain comparable to the wild-type level (S2 Fig), suggesting that DinB might be involved in replication past lesions specific to ImuC and *vice versa*. Thus, these results support our hypothesis that DinB is not able to protect cells against all the damage that accumulates in bacteria in the presence of MMS at 30°C (e.g. due to diminished efficiency of repair systems). As such, ImuC represents an essential backup polymerase to DinB following exposure to alkylating agents at low temperatures.

### ImuC is dispensable for the DNA alkylation damage tolerance in *P*. *putida* at 37°C

**Fig 12:**
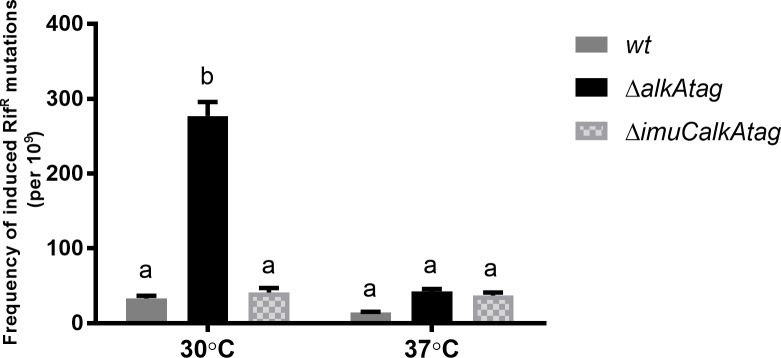
Effect of incubation temperature on the frequency of MMS-induced Rif^R^ mutations in *P*. *putida* strains. *P*. *putida* wild-type (*wt*), Δ*alkAtag* and Δ*imuCalkAtag* strains were incubated with 0.15 mM MMS overnight at 37°C or 30°C. The frequencies of MMS-induced mutagenesis were measured as described in Materials and Methods. Data represents the mean (±SE) values. Groups that have no common letter are significantly different at *P* < 0.0001, according to ANOVA followed by Bonferroni’s multiple comparisons test.

The effect of growth temperature on the survival of TLS polymerase-deficient *P*. *putida* strains in the presence of alkylation damage was different from that observed in the case of *P*. *aeruginosa*. When incubated at 30°C, the deficiency in either ImuC or DinB resulted in the increased MMS sensitivity of the Δ*alkA* cells, whereas the Δ*imuCdinBalkA* mutant was hypersensitive to DNA alkylation damage (Figs 4A and 9C). However, when bacteria were grown at 37°C, the effect of the *imuC* deletion became phenotypically inconspicuous, and only the loss of DinB resulted in a marked decrease in the survival of cells in the presence of MMS (Fig 9C). The absence of any survival defects following the deletion of *imuC* upon MMS exposure at 37°C encouraged us to conduct the MMS mutagenesis assay to evaluate the contribution of ImuC to the DNA alkylation lesion bypass at this temperature. In this study we demonstrated that the occurrence of MMS-induced mutations detected in the AlkATag-deficient *P*. *putida* at 30°C was largely dependent on the presence of ImuC (Fig 7). However, we unexpectedly uncovered that when bacteria were incubated at 37°C, the frequency of the MMS-induced mutations in the Δ*alkAtag* strain was about 6.5-fold lower in comparison to that measured at 30°C (Fig 12). Moreover, it was comparable to that observed in the Δ*imuCalkAtag* strain. This implies that at 37°C the DNA alkylation damage tolerance in *P*. *putida* is ImuC-independent.

**Fig 13:**
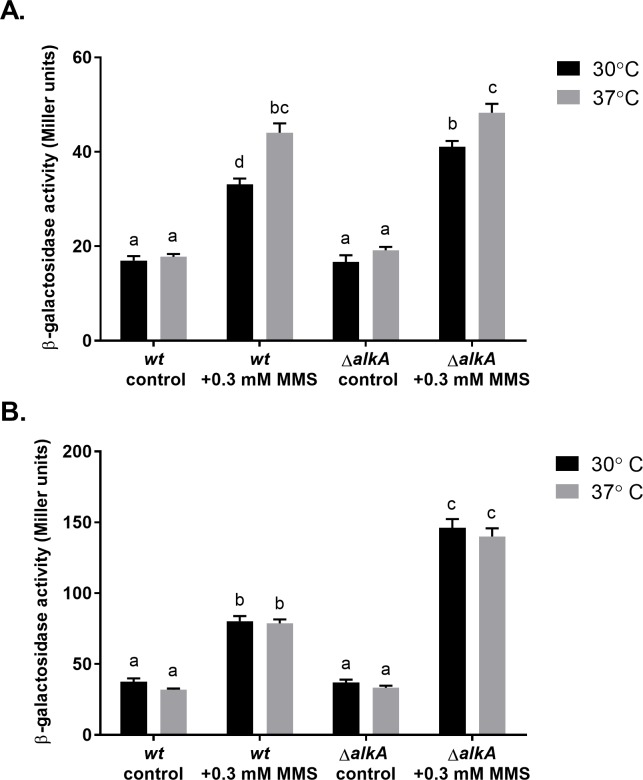
Effect of incubation temperature on transcription from the *P*. *putida lexA2* promoter. β-galactosidase activities expressed from the *lexA2* promoter-*lacZ* reporter were measured in the *P*. *putida* wild-type and in its *alkA-*deficient derivative incubated at 30°C (black) or 37°C (grey) for 1 hour (A) and overnight (B) in liquid LB medium supplemented with 0.3 mM MMS or not (control). Data represents the mean (±SE) values. Letters indicate homogeneous groups according to ANOVA followed by Bonferroni’s multiple comparisons test (*P* < 0.01).

In addition, when incubated at 37°C, the MMS tolerance of the *P*. *putida* AlkA-deficient strain was significantly higher (Fig 9C). The enhanced survival of bacteria at 37°C in the presence of MMS suggested that at this temperature the MMS-mediated damage could be lower (e.g., due to increased efflux of the chemical or enhanced repair efficiencies of alkylation damage). To address this question and assess the genotoxicity of MMS under different temperatures, we monitored the *lexA2* promoter activity in the transcriptional fusion with the *lacZ* reporter gene. In *P*. *putida* the *lexA2* gene is co-transcribed with the *imuABC* genes and the expression of the *lexA2-imuABC* transcriptional unit is upregulated upon DNA damage [42]. Thus, the *lexA2* transcriptional activity could represent *an indirect measure of* the DNA damage. We observed that the *β*-galactosidase activities from the *lexA2* promoter measured one hour after MMS exposure were significantly higher in the wild-type (*P* ≤ 0.0001) and in AlkA*-*deficient cells (*P* ≤ 0.01) incubated at 37°C (Fig 13A), which could be also attributed to a slight faster growth of bacteria. However, the *β*-galactosidase activities from this promoter measured in cells grown overnight in the presence of MMS were comparable at both temperatures (Fig 13B). These results indicate that the level of MMS-induced damage is similar at both growth temperatures. At the same time, the *lexA2* promoter activity in the MMS-treated Δ*alkA* strain was significantly higher (1.8 times, *P* ≤ 0.0001) than in the wild-type at both temperatures (Fig 13B), reflecting the higher degree of DNA damage in the bacteria lacking the AlkA*-*mediated repair of alkylated DNA. Thus, the increased survival of *P*. *putida* in the presence of MMS at 37°C is not related to a lower level MMS-induced DNA damage but could be a consequence of other temperature-influenced metabolic or physiological changes.

## Discussion

The significance of specialized DNA polymerase Pol V in *E*. *coli* in response to alkylation damage was demonstrated decades ago [39,40,67,69], whereas DNA damage tolerance mechanisms in bacteria that lack homologues of *umuDC* in their genomes mostly remained unexplored. These bacteria usually carry the damage-inducible multigene *imuABC* cassette [44], which encodes a TLS system responsible, for example, for MMC- and UV-induced mutagenesis in *M*. *tuberculosis* [46,47] and *C*. *crescentus* [45], and for UV-mutagenesis in *P*. *aeruginosa* [49]. In the present study, we revealed a possible role of the specialized DNA polymerases ImuC and DinB in DNA alkylation damage tolerance and their contribution to the MMS-induced mutagenesis in *P*. *aeruginosa* and *P*. *putida*.

Considering the number of potentially toxic DNA alkylation lesions induced by different methylating agents and individual DNA repair capacities, the survival strategy upon alkylation damage might be organism-specific [11]. Similarly to *E*. *coli* [38], we observed a critical role of DinB in the tolerance to MMS-induced damage in *P*. *aeruginosa* (Fig 1A). In addition, the increased sensitivity of *P*. *aeruginosa* strain deficient in both DinB and ImuC in comparison to that lacking DinB alone uncovered the importance of ImuC in the protection against cytotoxicity induced by MMS. At the same time, the survival of *P*. *putida* on MMS-containing plates was not affected when DinB, ImuC or both TLS DNA polymerases were absent (Fig 1C). However, the growth phase of bacteria affected the MMS-tolerance mechanisms. We found that the DNA alkylation damage tolerance was significantly reduced in the absence of DinB and ImuC when *P*. *putida* cells were grown exponentially in liquid cultures (Fig 2). The fact that MMS-induced damage on MMS-containing plates appeared to be toxic in *P*. *aeruginosa* but not in *P*. *putida* TLS-deficient strains highlights the differences in the DNA alkyl damage repair efficiencies between these species. Up to date there are only a few published reports about alkylation damage repair in *P*. *aeruginosa* [70,71]. However, it is known that *P*. *putida* strains vary not only in the *alkA* promoter induction or organization of the adaptive regulon but also harbour a different number of copies of genes encoding the DNA alkylation repair enzymes [66,72]. In addition, *P*. *aeruginosa* and *P*. *putida* possess AlkB proteins which belong to the different groups based on their sequence phylogeny [73]. Moreover, the genome of *P*. *putida* KT2440 harbours genes for at least four DNA glycosylases: *alkA* for damage-inducible DNA glycosylase II (AlkA); *tag*, for constitutively expressed DNA glycosylase I (Tag); an extra copy of the *tag* gene (*tag2*) and *pp_4812* gene, encoding for 3meA DNA glycosylase [66]. Therefore, it is possible that the absence of any phenotypical impact of the TLS polymerase deficiency upon MMS exposure in stationary *P*. *putida* cells might be associated with a rapid and efficient removal and repair of MMS-induced damage by the DNA glycosylase-mediated BER [66].

The effect of TLS polymerase deficiency on the survival of bacteria with impaired BER functions, lacking *alkA* and/or *tag* genes, allowed us to define more precisely the contribution of ImuC and DinB to alkylation damage response. The extreme MMS and MNNG sensitivity of mutants deficient in DNA glycosylases (Fig 3) in comparison with moderate sensitivity of TLS deficient strains (Fig 1) demonstrated that DNA-glycosylase initiated BER is the main mechanism that protects cells against alkylation damage, while TLS has a secondary role here. At the same time, both ImuC and DinB appeared to be critical for the protection of *alkA* and/or *tag* bacteria against alkylation damage that accumulates in cells deficient in DNA glycosylase-mediated repair (Figs 4 and 5). It is known that *E*. *coli* DNA glycosylase-deficient cells are DNA alkylation sensitive due to the lack of 3meA and 3meG repair [4]. However, the alkylation damage sensitivity of pseudomonads (at least of *P*. *putida*) with impaired glycosylase activity is more likely attributable also to 1meA and 3meC that are substrates for AlkB and AlkA in *P*. *putida* [66]. As such, we suggest that ImuC and DinB could be involved in the TLS past MMS-generated 3meA, 3meG, 1meA and 3meC lesions. In addition, the specificity of alkylation lesion bypass by ImuC and DinB might be related in both species, because the survival profile of *P*. *aeruginosa* AlkA-deficient cells lacking ImuC or DinB upon alkylation damage was similar to the corresponding *P*. *putida* strains (Fig 4A and 4B; Fig 5A and 5B).

But do DinB and ImuC replicate past the same DNA lesions or does their specificity differ? Considering that ImuC involvement in alkylation damage response was observed in bacteria lacking DinB, we suggest that ImuC may act as a secondary polymerase to DinB. Moreover, the overexpression of DinB in Δ*imuC* and Δ*imuCdinB* strains increased the MMS tolerance of these mutants to the wild-type level, suggesting that DinB could carry out TLS past some of the lesions bypassed by ImuC. However, hypersensitivity of *P*. *putida* AlkA and AlkATag-deficient cells as well as *P*. *aeruginosa* AlkA-deficient and -proficient bacteria in the absence of both TLS polymerases suggests that ImuC and DinB could protect cells against different DNA lesions.

In addition to methylated bases, MMS promotes the production of lethal abasic sites (AP) either by excision of alkylation products by DNA glycosylases or by spontaneous depurination of unstable *N*-methylpurines [3,74]. The toxicity resulting from 7meG is not significant because of a very slow depurination rate (half-life is app. 150h) [75,76]. However, less stable 3meA (half-life ranging from 4 to 24 h and 40-fold shorter in ssDNA) [9,75,77] significantly contributes to the appearance of replication-blocking AP sites that may lead to the formation of double strand breaks (DSB) and subsequent cell death [77]. Both Pol V and Pol IV are able to bypass AP sites *in vitro* in *E*. *coli*, while Pol V plays a major role in the replication across AP sites *in vivo* [78–81]. Thus, by drawing parallels with these data, it is possible that in pseudomonads the ImuC- and DinB-dependent replication past AP sites could also facilitate survival of cells upon MMS treatment. Furthermore, if MMS predominantly produces *N*-methylation, then MNNG generates both *N*- and *O*-methylated bases [1]. The O^6^meG lesion is thought to be replication-blocking in *E*. *coli* [82]. The notion that ImuC appeared to have more critical role than DinB in the protection of AlkA-deficient *P*. *putida* and *P*. *aeruginosa* bacteria against MNNG-induced damage (Figs 4B and 5B) consequently suggests a potential association of ImuC with the TLS pathway or repair of O^6^meG lesions.

Our results demonstrate that the mutability of MMS in *P*. *putida* and *P*. *aeruginosa* depends on ImuC functions (Figs 7 and 8). On the other hand, the accurate bypass of alkylation damage by DinB detected in both pseudomonads *in vivo*, similarly to that in *E*. *coli*, supports the evolutionary conservation of Pol IV functions among different species of bacteria [38]. Thus, DinB in pseudomonads might also act as a restrictive factor of the mutagenic property of ImuC-mediated TLS, similarly to its actions in *E*. *coli*, where the presence of DinB was shown to minimize the Pol V-dependent mutagenic replication past AP sites [38,83]. Recent study in *E*. *coli* demonstrated that during the stress response DinB and RecA are able to slow down the replication fork progression [84], thereby providing additional time for DNA repair. Thus, it is possible that either direct (accurate TLS and competing with ImuC for lesion bypass) or indirect (inhibition of replication fork progression) properties of DinB might limit the alkylation damage-induced mutagenesis and mediate protection of the genome integrity.

Notably, we show for the first time that the incubation temperature of bacteria appears to be an important factor determining the implication of TLS in the alkylation damage response in *Pseudomonas* species. In regards to temperature, bacteria modulate gene expression profile, leading to alterations in membrane composition, metabolism, replication and other general adaptive responses [85,86]. In the current study, the requirement of ImuC for the protection of *P*. *aeruginosa* against MMS cytotoxicity appeared to be more critical at the temperature below the growth optimum (Fig 9A and 9B). For example, in wild-type background the deletion of ImuC, which was phenotypically undetectable upon MMS treatment at 37°C, led to the decreased survival at 30°C (Figs 1A and 9A). Because of the slower growth at 30°C leading to prolonged incubation on chemical-supplemented plates, we first assumed that the increased sensitivity of cells lacking ImuC might be associated with toxicity resulting from the higher stability of 1meA and 3meC lesions that become more relevant in time, if compared to quite unstable 3meA lesions [87]. However, the decreased survival of MMS-treated ImuC-deficient bacteria after overnight incubation at lower temperature did not support that hypothesis (Fig 10). Unexpectedly, in MMS-treated bacteria incubated at 30°C the transcriptional activity of the *dinB* gene was significantly higher than in cells incubated at 37°C (Fig 11). Since *dinB* gene is DNA-damage inducible, our results suggest that MMS-induced damage at 30°C might be higher. This could be associated with the decreased efficiency or different regulation of DNA repair (including BER) and/or damage tolerance systems that provide protection against alkylation damage. Thus, under such conditions DinB seems to be unable to protect cells against accumulating damage and as a consequence of the increased need for ImuC in promoting survival, cells lacking ImuC-mediated activities become more sensitive to the alkylating agents.

In the case of *P*. *putida*, the shift in growth temperature from 30°C to 37°C resulted in the disappearance of ImuC-dependent MMS tolerance- and mutator phenotypes (Figs 9C and 12). In addition to metabolic or physiological changes associated with modulation of gene expression, the growth temperature directly affects the topology and the structure of DNA, protein folding and stability, protein-DNA and protein-protein interactions [88,89]. As was previously demonstrated in *M*. *tuberculosis* [46] and in *C*. *crescentus* [45], and also supported by the results of the present study in pseudomonads (Fig 6), all three products of the *imuABC* operon are critical for proper functioning of the TLS system. Thus, one may speculate that the functionality of the ImuABC-mediated TLS in *P*. *putida* might be affected by the temperature via complex stability or its interactions with DNA and other proteins, resulting in the disappearance of ImuC-dependent phenotypes at higher temperatures. However, further investigations are needed to confirm this hypothesis.

In conclusion, our study corroborates the evolutionary significance of DinB in protection of cells and genome stability upon alkylation damage. Moreover, we uncover the role of ImuC in alkylation damage tolerance in bacteria that lack Pol V. Despite the fact that the impact of ImuC on the cell survival in the presence of alkylating agents was observed under DinB-deficient conditions and in bacteria with impaired BER functions due to AlkA/Tag deficiency, the importance of ImuC might be associated with its error-prone activity, which can be beneficial under stressful conditions. In addition, our results reveal the importance of growth temperature in pseudomonads that can directly or indirectly affect the TLS function in response to alkylation DNA damage. This demonstrates how changes in the environment modulate the cellular damage responses and overall complexity of cellular processes and systems.

## Supporting Information

S1 Fig MMS sensitivity of *P*. *putida* Δ*alkA* strain, its *imuB*-deficient derivative and *alkAimuB*-deficient mutant complemented with *imuB* inserted into *att*Tn*7* sites. Sensitivity to MMS was estimated by spotting 10-fold dilutions of mid-exponential cultures onto LB plates containing 0.5 mM MMS, supplemented with IPTG (B) or not (A), and incubated at 30°C for 24 h.
(TIF)

S2 Fig MMS sensitivity of *imuC*-, *dinB*- and *imuCdinB* deficient *P*. *aeruginosa* strains carrying PJB vector with *dinB* gene under the control of *P*_*tac*_ promoter. Sensitivity to MMS was estimated by spotting 10-fold dilutions of overnight cultures onto LB plates containing 3 mM MMS, supplemented with IPTG (B) or not (A), and incubated at 30°C for 48 h.
(TIF)

S1 Table Bacterial strains and plasmids. (DOCX)

S2 Table Oligonucleotides used in the study. (DOCX)
