# Supplementary figures and images for: DNA Polymerases ImuC and DinB Are Involved in DNA Alkylation Damage Tolerance in Pseudomonas aeruginosa and Pseudomonas putida
Source: PLoS One. 2017 Jan 24;12(1):e0170719. doi: 10.1371/journal.pone.0170719 (PMC5261740; doi:10.1371/journal.pone.0170719)

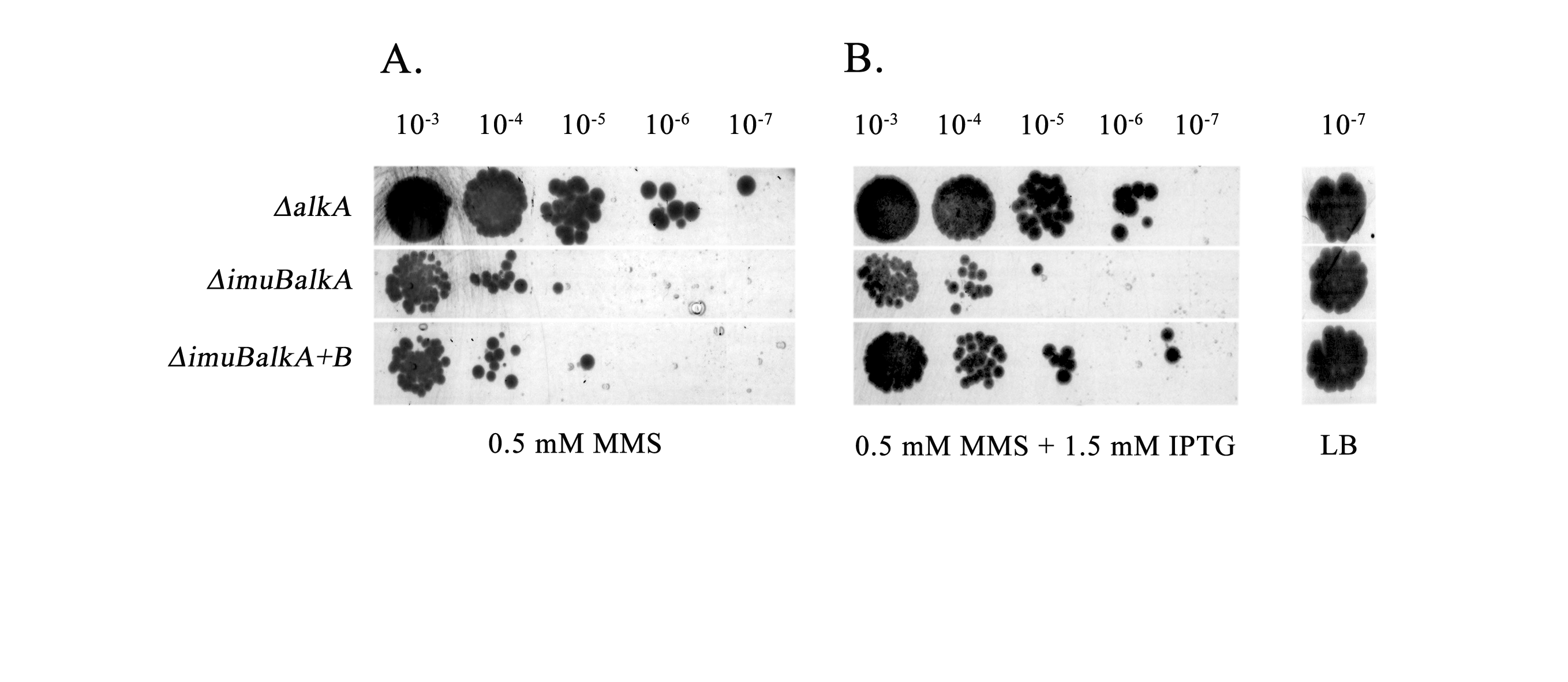

Supplement: S1 Fig — Sensitivity to MMS was estimated by spotting 10-fold dilutions of mid-exponential cultures onto LB plates containing 0.5 mM MMS, supplemented with IPTG (B) or not (A), and incubated at 30°C for 24 h. (TIF) [file pone.0170719.s001.tif]

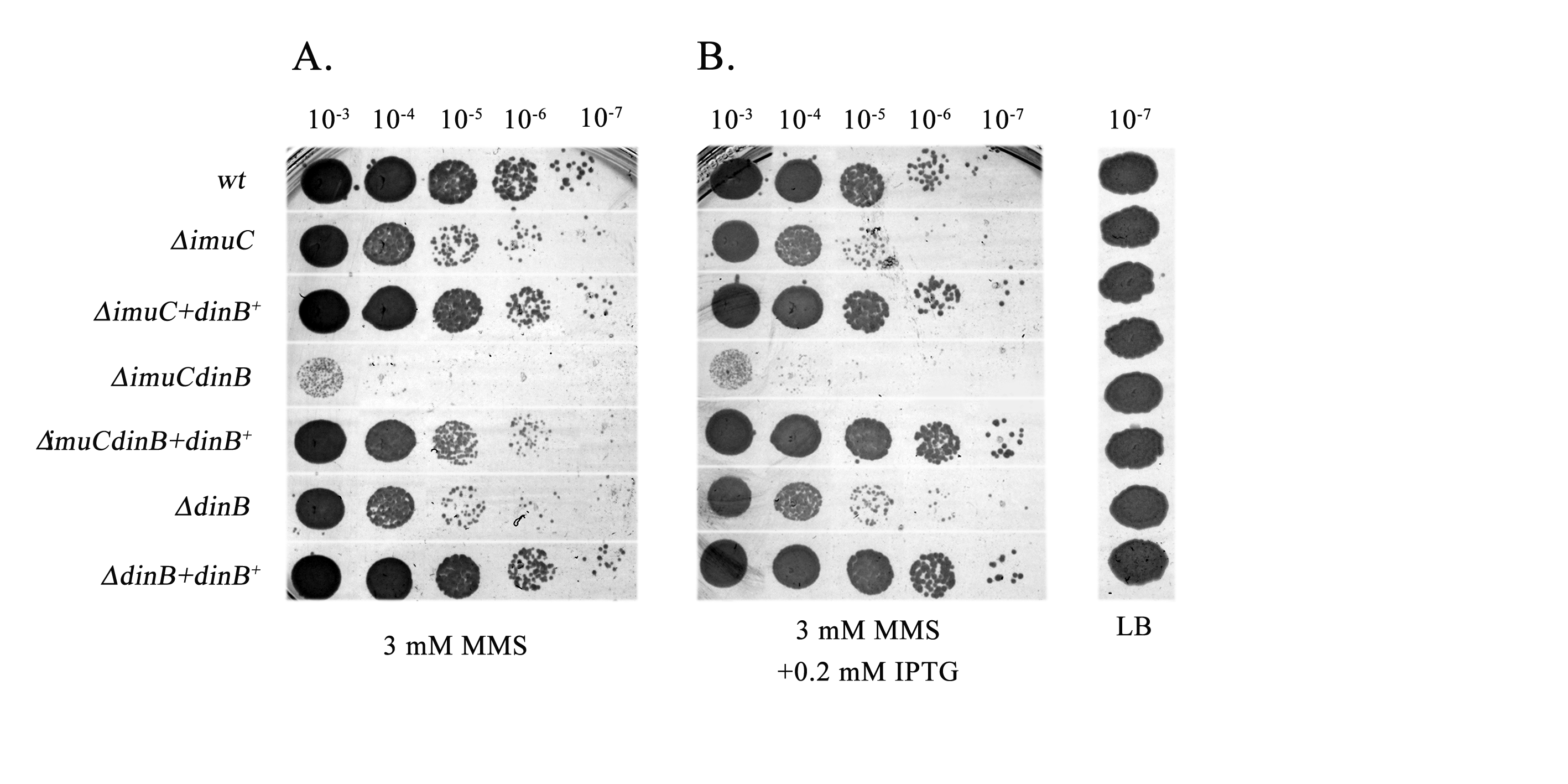

Supplement: S2 Fig — Sensitivity to MMS was estimated by spotting 10-fold dilutions of overnight cultures onto LB plates containing 3 mM MMS, supplemented with IPTG (B) or not (A), and incubated at 30°C for 48 h. (TIF) [file pone.0170719.s002.tif]
