# Supplementary material for: DNA Polymerases ImuC and DinB Are Involved in DNA Alkylation Damage Tolerance in Pseudomonas aeruginosa and Pseudomonas putida
Source: PLoS One. 2017 Jan 24;12(1):e0170719. doi: 10.1371/journal.pone.0170719 (PMC5261740; doi:10.1371/journal.pone.0170719)
Supplement: S1 Table — (DOCX) [file pone.0170719.s003.docx]

**S1 Table. Bacterial strains and plasmids.**

| Strain or plasmid | Genotype or charasteristics | Source or reference |
| --- | --- | --- |
| *E. coli* strains |  |  |
| DH5α | supE44 ΔlacU169 (Φ80 lacZΔM15) recA1 endA1 hsdR17 thi-1 gyrA96 relA1 | [1] |
| DH5α λpir | λ*pir lysogen of DH5* α | [2] |
| CC118 λ*pir* | Δ*(ara-leu) araD* Δ*lacX74 galE galK phoA20 thi-1 rpsE rpoB argE(Am) recA1 λpir lysogen* | [3] |
| *P. putida* strain or genotype |  |  |
| PaW85 | Wild-type, isogenic to KT2440 | [4] |
| PpΔ*imuC* | PaW85, Δ*imuC* (ΔPP3119) | This study |
| PpΔ*dinB* | PaW85, Δ*dinB* (ΔPP1203) | This study |
| PpΔ*imuCdinB* | PaW85, Δ*imuC;* Δ*dinB* | This study |
| PaWAlkA | PaW85, Δ*alkA* | [5] |
| PpΔ*imuAalkA* | PaW85, Δ*alkA;* Δ*imuA* (PP3117) | This study |
| PpΔ*imuBalkA* | PaW85, Δ*alkA;* Δ*imuB* (PP3118) | This study |
| PpΔ*imuCalkA* | PaW85, Δ*alkA;* Δ*imuC* | This study |
| PpΔ*imuABCalkA* | PaW85, Δ*alkA;* Δ*imuA;* Δ*imuB;* Δ*imuC* | This study |
| PpΔ*imuBalkA+B* | PaW85, Δ*alkA;* Δ*imuB; att*Tn*7*::miniTn*7*-*imuB,* Sm*^r^* | This study |
| PpΔ*dinBalkA* | PaW85, Δ*alkA;* Δ*dinB* | This study |
| PpΔ*imuCdinBalkA* | PaW85, Δ*alkA;* Δ*imuC,* Δ*dinB* | This study |
| PpΔ*tag* | PaW85, Δ*tag* (ΔPP0062) | This study |
| PpΔ*imuCtag* | PaW85, Δ*tag;* Δ*imuC* | This study |
| PpΔ*dinBtag* | PaW85, Δ*tag;* Δ*dinB* | This study |
| PpΔ*imuCdinBtag* | PaW85, Δ*tag;* Δ*imuC;* Δ*dinB* | This study |
| PpΔ*alkAtag* | PaW85, Δ*alkA;* Δ*tag* | This study |
| PpΔ*imuCalkAtag* | PaW85, Δ*alkA;* Δ*tag,* Δ*imuC* | This study |
| PpΔ*dinBalkAtag* | PaW85, Δ*alkA;* Δ*tag;* Δ*dinB* | This study |
| PpΔ*imuCdinBalkAtag* | PaW85, Δ*alkA;* Δ*tag; imuC;* Δ*dinB* | This study |
| *P. aeruginosa* strain or genotype | | |
| PAO1-L | PAO1 subline, University of Lausanne, Dieter Haas collection | Stephan Heeb |
| PaΔ*imuC* | PAO1-L, Δ*imuC* (ΔPA0669) | This study |
| PaΔ*dinB* | PAO1-L, Δ*dinB* (ΔPA0923) | [6] |
| PaΔ*imuCdinB* | PAO1-L, Δ*imuC;* Δ*dinB* | This study |
| PaΔ*alkA* | PAO1-L, Δ*alkA* (ΔPA1686) | This study |
| PaΔ*imuAalkA* | PAO1-L, Δ*alkA;* Δ*imuA* (ΔPA0671) | This study |
| PaΔ*imuBalkA* | PAO1-L, Δ*alkA;* Δ*imuB* (ΔPA0670) | This study |
| PaΔ*imuCalkA* | PAO1-L, Δ*alkA;* Δ*imuC* | This study |
| PaΔ*imuABCalkA* | PAO1-L, Δ*alkA;* Δ*imuA;* Δ*imuB;* Δ*imuC* | This study |
| PaΔ*dinBalkA* | PAO1-L, Δ*alkA;* Δ*dinB* | This study |
| PaΔ*imuCdinBalkA* | PAO1-L, Δ*alkA;* Δ*imuC,* Δ*dinB* | This study |
| Plasmids |  |  |
| pEMG | Suicide plasmid containing *lacZα* with two flanking I-SceI sites (Km^r^) | [2] |
| pSW(I-SceI) | Plasmid for I-SceI expression (Amp^r^) | [7] |
| pRK2013 | Helper plasmid for conjugal transfer (Km^r^) | [8] |
| pEMGPpDinB | pEMG containing a PCR-designed 1,1-kb Acc65I-XbaI insert for deleting *P. putida dinB* (PP1203) (Km^r^) | This study |
| pEMGPpImuC | pEMG containing a PCR-designed 1,1-kb BamHI-XbaI insert for deleting *P. putida imuC* (PP3119) (Km^r^) | This study |
| pEMGPpImuA | pEMG containing a PCR-designed 1,3-kb BamHI-XbaI insert for deleting *P. putida imuA* (PP3117) (Km^r^) | This study |
| pEMGPpImuB | pEMG containing a PCR-designed 1,2-kb BamHI-XbaI insert for deleting *P. putida imuB* (PP3118) (Km^r^) | This study |
| pEMGPpImuABC | pEMG containing a PCR-designed 1,2-kb BamHI-XbaI insert for deleting *P. putida imuABC* (PP3117-PP3119) (Km^r^) | This study |
| pEMGPpAlkA | pEMG containing PCR-amplified sequence of *P. putida alkA* in SacI and XbaI sites | [5] |
| pEMGPpTag | pEMG containing a PCR-designed 1,2-kb SacI-XbaI insert for deleting *P. putida tag* (PP0062) (Km^r^) | This study |
| pEMGPaDinB | pEMG containing a PCR-designed 1,1-kb SacI-XbaI insert for deleting *P. aeruginosa dinB* (PA0923) (Km^r^) | [6] |
| pEMGPaImuA | pEMG containing a PCR-designed 1,65-kb BamHI-XbaI insert for deleting *P. aeruginosa imuA* (PA0671) (Km^r^) | This study |
| pEMGPaImuB | pEMG containing a PCR-designed 1,4-kb SacI-XbaI insert for deleting *P. aeruginosa imuB* (PA0670) (Km^r^) | This study |
| pEMGPaImuC | pEMG containing a PCR-designed 1,1-kb SacI-XbaI insert for deleting *P. aeruginosa imuC* (PA0669) (Km^r^) | This study |
| pEMGPaImuABC | pEMG containing a PCR-designed 1,1-kb BamHI-XbaI insert for deleting *P. aeruginosa imuABC* (Km^r^) | This study |
| pEMGPaAlkA | pEMG containing a PCR-designed 1,2-kb SacI-XbaI insert for deleting *P. aeruginosa alkA* (PA1686) (Km^r^) | This study |
| p9TT_B_lacZ | Promoter probe plasmid (Cm^r^, Ap^r^) | [9] |
| p9TT_B_lacZ-lexA2 | p9TT_B_lacZ containing the promotor region of *lexA2* cloned upstream of lacZ gene in BamHI (Ap^r^) | This study |
| pUCNotlacItac | Expression vector containing P*_tac_* promoter and *lacI*^q^ repressor (Ap^r^) | Rita Hõrak |
| pUCNotlacItac-ImuB | pUCNotlacItac containing *imuB* under the control of *lacI* and P*_tac_* promoter in SmaI (Ap^r^) | This study |
| pBK-miniTn*7*-ΩSm1 | pUC19-based delivery plasmid for mini-Tn*7*-ΩSm1, (Ap^r^, Sm^r^, *mob^+^)* | [10] |
| pBK-miniTn*7*-ImuB | pBK-miniTn*7*-ΩSm1 containing *lacI*^q^*-P_tac_*-*imuB* expression cassette from pUCNotlacItac-*imuB* in KpnI and SmaI (Ap^r^, Sm^r^) | This study |
| pBLKT | Promoter probe vector (Km^r^) | [11] |
| pBLKT-PAdinB | Promoter region of *dinB* gene cloned into pBLKT BamHI site (Km^r^) | This study |
| pJB-*lacI*^q^-P*_tac_* | Expression vector containing P*_tac_* promoter and *lacI*^q^ repressor (Ap^r^) | [12] |
| pJB-PadinB | pJB-*lacI*^q^-P*_tac_* containing *P. aeruginosa* *dinB* under control of P*_tac_* promoter in XbaI and BglII (Ap^r^) |  |

[1] D. Hanahan, M. Meselson, Plasmid screening at high colony density., Gene. 10 (1980) 63–67.

[2] E. Martínez-García, V. de Lorenzo, Engineering multiple genomic deletions in Gram-negative bacteria: analysis of the multi-resistant antibiotic profile of *Pseudomonas putida* KT2440., Environ. Microbiol. 13 (2011) 2702–16. doi:10.1111/j.1462-2920.2011.02538.x.

[3] M. Herrero, V. de Lorenzo, K.N. Timmis, Transposon vectors containing non-antibiotic resistance selection markers for cloning and stable chromosomal insertion of foreign genes in gram-negative bacteria., J. Bacteriol. 172 (1990) 6557–67.

[4] S.A. Bayley, C.J. Duggleby, M.J. Worsey, P.A. Williams, K.G. Hardy, P. Broda, Two modes of loss of the tol function from *Pseudomonas putida mt-2*, MGG Mol. Gen. Genet. 154 (1977) 203–204.

[5] D. Mielecki, S. Saumaa, M. Wrzesiński, A.M. Maciejewska, K. Żuchniewicz, A. Sikora, et al., *Pseudomonas putida* AlkA and AlkB Proteins Comprise Different Defense Systems for the Repair of Alkylation Damage to DNA – In Vivo, In Vitro, and In Silico Studies, PLoS One. 8 (2013). doi:10.1371/journal.pone.0076198.

[6] J. Sidorenko, Combating DNA damage and maintenance of genome integrity in pseudomonads, 2015.

[7] S.M. Wong, J.J. Mekalanos, Genetic footprinting with mariner-based transposition in *Pseudomonas aeruginosa*., Proc. Natl. Acad. Sci. U. S. A. 97 (2000) 10191–10196.

[8] D.H. Figurski, D.R. Helinski, Replication of an origin-containing derivative of plasmid RK2 dependent on a plasmid function provided in trans., Proc. Natl. Acad. Sci. U. S. A. 76 (1979) 1648–52.

[9] P.A. Kivistik, M. Putrins, K. Püvi, H. Ilves, M. Kivisaar, R. Hõrak, The ColRS two-component system regulates membrane functions and protects *Pseudomonas putida* against phenol., J. Bacteriol. 188 (2006) 8109–17. doi:10.1128/JB.01262-06.

[10] B. Koch, L.E. Jensen, O. Nybroe, A panel of Tn7-based vectors for insertion of the gfp marker gene or for delivery of cloned DNA into Gram-negative bacteria at a neutral chromosomal site, J. Microbiol. Methods. 45 (2001) 187–195. doi:10.1016/S0167-7012(01)00246-9.

[11] A. Lahesaare, H. Moor, M. Kivisaar, R. Teras, I. Sutherland, L. Friedman, et al., *Pseudomonas putida* Fis Binds to the lapF Promoter In Vitro and Represses the Expression of LapF, PLoS One. 9 (2014) e115901. doi:10.1371/journal.pone.0115901.

[12] J. Jakovleva, A. Teppo, A. Velts, S. Saumaa, H. Moor, M. Kivisaar, et al., Fis regulates the competitiveness of *Pseudomonas putida* on barley roots by inducing biofilm formation, Microbiology. 158 (2012) 708–720. doi:10.1099/mic.0.053355-0.
