# Supplementary material for: DNA Polymerases ImuC and DinB Are Involved in DNA Alkylation Damage Tolerance in Pseudomonas aeruginosa and Pseudomonas putida
Source: PLoS One. 2017 Jan 24;12(1):e0170719. doi: 10.1371/journal.pone.0170719 (PMC5261740; doi:10.1371/journal.pone.0170719)
Supplement: S2 Table — (DOCX) [file pone.0170719.s004.docx]

**S2 Table. Oligonucleotides used in the study**

| Primer name | Sequence (5’→3’) | Restriction site |
| --- | --- | --- |
| PpDinB_Ts1_F_Acc65I | ATA TGG TAC CCG AAT GCT TAT CAG CAG GCA A | Acc65I |
| PpDinB_Ts1_R | TTC ATT TGG GCG GAA ACA GCG ACC GTC AGC ACT GTA AAT TC |  |
| PpDinB_Ts2_F | GCT GTT TCC GCC CAA ATG AA |  |
| PpDinB_Ts2_R_XbaI | ATA TTC TAG ACA TGA ACG TGC CAT CCA ACG | XbaI |
| PpImuA_Ts1_F_BamH | ATA TGG ATC CGG GCT GTT CAG CGT GCT CCG AA | BamHI |
| PpImuA_Ts1_R | CAG GCC CAG AGC ATG GTG CAT CAG TCG CGC CTC AGC AAG |  |
| PpImuA_Ts2_F | TGC ACC ATG CTC TGG GCC TGC A |  |
| PpImuA_Ts2_R_Xba | ATA TTC TAG AAT CGC GCG GCA AGG CCA ATA C | XbaI |
| PpImuB_Ts1_F_BamH | ATA TGG ATC CAC GAA CGT TTG CCG GAA GGT G | BamH |
| PpImuB_Ts1_R | GGG TTT CGA TGC GGT AGT AGT GGT GCA TCA GCC CCG CCC |  |
| PpImuB_Ts2_F | ACT ACT ACC GCA TCG AAA CCC |  |
| PpImuB_Ts2_R_XbaI | ATA TTC TAG ACG GAT GGC AGT CAT GCA GTC | XbaI |
| PpImuC_Ts1_F_BamH | ATA TGG ATC CCG GAT ACC CTG CTC AAG GTG G | BamHI |
| PpImuC_Ts1_R | AGT ACC GAG CGT TGG GGT TCA ATG CAG CTC GGC ATA ACC |  |
| PpImuC_Ts2_F | AAC CCC AAC GCT CGG TAC T |  |
| PpImuC_Ts2_R_XbaI | ATA TTC TAG ATT CGA CTT GCT ACC GTG GTG | XbaI |
| PpImuABC_Ts1_R | AGT ACC GAG CGT TGG GGT TTC AGT CGC GCC TCA GCA AGC |  |
| PpTag_Ts1_F | ATA TGA GCT CAC ACC AGG TCG TAC ACC G | SacI |
| PpTag_Ts1_R | TCA GCA AGG CGC GCA TTG CAG GTT ATA CTC CCG CTC T |  |
| PpTag_Ts2_F | CAA TGC GCG CCT TGC TGA |  |
| PpTag_Ts2_R | ATA TTC TAG AGA ACG GTA CGA ACA CGC C | XbaI |
| PaAlkA_Ts1_F | ATA GAG CTC TCG GTG GAG TCG GAG ATG AT | SacI |
| PaAlkA_Ts1_R | CAA CGT CAG GCT GTA GCC TCC GGG CAG ACG ATA CCT GTC CG |  |
| PaAlkA_Ts2_F | GGA GGC TAC AGC CTG ACG TT |  |
| PaAlkA_Ts2_R | ATA TTC TAG ATC AGT TGA AAG GCC ACG TCT | XbaI |
| PaImuC_Ts1_R | AGG CCG GTG AGT AGA GGG GTT CAT GCG AAC CAG CCA TGC AG |  |
| PaImuC_Ts1_F | TAT GAG CTC CTG CTC TTC CCC CTG CGA CG | SacI |
| PaImuC_Ts2_F | ACC CCT CTA CTC ACC GGC CT |  |
| PaImuC_Ts2_R | ATA TCT AGA CGT AAG CCC AAG CGA ACG GA | XbaI |
| PaImuA_TS2_F | CGC ATC CTC AAG TGC CG |  |
| PaImuA_TS2_R_ XbaI | ATA TTC TAG ACA ACT GTT CCC AGC CGA GAT | XbaI |
| PaImuA_TS1_R | CGG CAC TTG AGG ATG CGG GCG GAC TGG CCT TTC CA |  |
| PaImuA_TS1_F_BamH | ATA TGG ATC CCA GCG CTT CGT TGC GAT AAA | BamHI |
| PaImuB_Ts1_F | TAT GAG CTC CTC GAC GCA CGT AGG ATC TG | SacI |
| PaImuB_Ts1_R | GCT ACC GAC CTC GCG ATA GAC AGA GCA TGG CCG CCT CAT T |  |
| PaImuB_Ts2_F | TCT ATC GCG AGG TCG GTA GC |  |
| PaImuB_Ts2_R | ATA TCT AGA ATC TTC AGG GTT TCC GCC AG | XbaI |
| PaImuABC_Ts1_R | AGG CCG GTG AGT AGA GGG GTG GCG GAC TGG CCT TTC CA |  |
| LexA2-140BamH | ATA TGG ATC CGC AGG GTG TCA GTG TCG GTG AG | BamHI |
| LexA2+92BamH | ATA TTG GAT CCA AAA CCG AAG CGC GTG GCG A | BamHI |
| dinb2lookus | TGG CAT GCC ACC AGC GCT |  |
| dinb2alamus | TTA TGC GAA CCA ACC CTG CAG CCA AAG |  |
| PaoDinB_prom_F_BamH | ATA TGG ATC CGA GTT CCA TCC GGT TCA CGC | BamHI |
| PaoDinB_prom_R_BamH | ATA TGG ATC CTA GCT GCA AGT GGC GAC C | BamHI |
| PAO_dinBalu_BglII | ATA TAG ATC TGC ATC AGA ACA ACC TGA GTT | BglII |
| PAO_dinB_RBS | ATA TTC TAG AAG GGG GGA TTC CGG TGC GGA AAA TC | XbaI |

The underlined sequences indicate restriction enzyme sites.
